# How do people in China think about causes of their back pain? A predominantly qualitative cross-sectional survey

**DOI:** 10.1186/s12891-020-03500-1

**Published:** 2020-07-21

**Authors:** YiJun Li, Michel W. Coppieters, Jenny Setchell, Paul W. Hodges, Gwendolyne G. M. Scholten-Peeters

**Affiliations:** 1 Amsterdam Movement Sciences, Faculty of Behavioural and Movement Sciences, Vrije Universiteit Amsterdam, Van der Boechorststraat 9, Amsterdam, 1081 BT The Netherlands; 2 Menzies Health Institute Queensland, Griffith University, Brisbane and Gold Coast, Australia; 3 School of Health and Rehabilitation Sciences, The University of Queensland, Brisbane, Australia

**Keywords:** Lumbar, Pain beliefs, Pain perception, China, Cultural sensitivity, Discourse analysis, Thinking patterns, Psychosocial, Rehabilitation, Disability

## Abstract

**Background:**

Low back pain (LBP) is the second highest cause of health burden in China. Delayed recovery, poor clinical outcomes and persistence of LBP are associated with negative pain beliefs about LBP. Chinese philosophies are nested into the daily life of people in China, which is likely to influence pain beliefs. However, there is lack of knowledge about people’s discourses regarding their LBP in China. The primary aim of this study was to explore the discourses underlying the beliefs of people in China about what causes their persistent or recurrent LBP. The secondary aim was to investigate the sources of these pain beliefs.

**Methods:**

People (*n* = 152) from South Central, East and North Mainland China with LBP completed an online survey about what they believed caused their persistent or recurrent LBP and where these understandings came from. Potential causes of persistent or recurrent LBP were explored qualitatively using discourse analysis. The sources of these discourses were assessed by descriptive statistics with conventional content analysis.

**Results:**

Five discourses were identified to underpin participants’ beliefs about what caused their persistent or recurrent LBP, namely: (1) biomedical problems (66.4%), (2) unbalanced lifestyle (48.7%), (3) menstruation and ‘kidney’ status (9.2%), (4) the ‘Five Elements’ imbalance (7.9%), and (5) energy status (5.9%). Most participants responded that their pain beliefs were based on information derived from healthcare professionals (59.2%), followed by the internet (24.3%) and family (23.0%).

**Conclusions:**

People from moderately and well-developed parts of Mainland China think predominantly in line with a Western biomedical viewpoint about their LBP. Traditional Chinese medicine related pain beliefs mainly to the concept of ‘balance’ were evident on contemporary Chinese society’s understandings of LBP. These cultural beliefs could be relevant to consider in LBP management and involve healthcare professionals, family and patient in this process.

## Background

Low back pain (LBP) is a common and disabling disorder, ranked as the leading cause of Years Lived with Disability (YLDs) worldwide [1, 2]. In China, a country with a population of 1.4 billion people [3], LBP was estimated to impact 67.3 million people in 2016 with an increase of 19% since 1990 [4], and is ranked as the second highest reason for health burden [4]. The annual prevalence of LBP varies between different occupations [5–8] ranging from 40% in teachers [5] to 74% in garment workers [5]. Considering that the working population reported 2.5 times higher persistent LBP than non-working population in low and middle-income regions [9], China, the biggest labour force country [10], suffers high economic and societal burden of LBP.

The medical service system in China consists of primary medical services (e.g., community healthcare centres and clinics) and secondary/tertiary hospitals [11]. Western medicine oriented hospitals are dominant in China but many of them have a traditional Chinese medicine department [11]. Each city or county has at least one traditional Chinese medicine hospital [11]. Referrals are not required [12], which means that all patients have direct access to healthcare. Diagnostic imaging is prescribed on a self-service basis or by various healthcare professionals. The excessive diagnostic imaging for LBP in China adds considerable burden on healthcare in China but is also associated with assumptions that diagnostic imaging could identify the causes of LBP [13].

For nearly all people with LBP, it is currently not possible to identify the specific nociceptive source [14]. Intervertebral discs, facet joints and vertebral endplate abnormalities are considered as potential nociceptive contributors to LBP [15–17]. There is now considerable research, predominantly conducted in the West, that suggests that attributing LBP to an anatomical basis has an important influence on patients’ beliefs. These pain related beliefs are considered to be formed by an individual’s past experience of pain and healthcare [18] as well as cultural influences [19]. There is also a large body of research which suggests that there are psychological effects of such biological pain beliefs such as fear avoidance, low self-efficacy and pain catastrophizing beliefs which are related to delayed recovery, poor clinical outcome and chronicity of LBP [20–23]. Associations between anatomical/ biomechanical pain beliefs and greater fear avoidance beliefs and correspondingly poor clinical outcomes have been found [24]. However, again, the current knowledge about these LBP beliefs [20–23] or beliefs about causes of pain [25–30] mainly comes from high-income countries, most with Western-medical belief systems. Whether this knowledge would also be applicable to low and middle-income countries such as China [31], and those with different systems of belief requires study [32]. A small amount of research has already suggested that pain beliefs are likely to vary between cultures, races, ethnicities, healthcare and countries [33–36].

A recent study reported four discourses (patterns of ideas) underlying beliefs of Australians about the causes of persistent or recurrent LBP [30]. The discourses were predominantly biomechanical or anatomical, which is in line with the traditional western biomedical view of health [30]. Comparable biomedical beliefs about the cause of LBP were found in another study attributing pain to anatomical vulnerability of the spine [35]. As Chinese philosophies underpin people’s daily life in China, it is likely that these philosophies would influence thinking about health for people living in China [37]. For instance, Yin-Yang posits that the development of a disease is caused by the imbalance of equilibrium in the body [37–39]. This ideology suggests that equilibrium can be disrupted by various factors, such as an excess of particular emotions (e.g., anger, happiness), overload (e.g., mental and physical), dietary imbalance and also by climate/weather (e.g., wind, cold weather) [40]. Although Western medicine is commonly practiced in China, it is plausible that Chinese philosophies also underpin beliefs about pain and persistence of pain. The discourses underpinning peoples’ beliefs about the causes persistent or recurrent LBP for people living in China have not yet been investigated and are important for improving clinical pain management. Understanding patients’ beliefs helps us to develop interventions addressing unhelpful beliefs in patients with LBP, and to target those interventions to patients who are most likely to benefit [26]. Successfully modifying patients’ pain beliefs might prevent delayed recovery and poor outcome [26]. Therefore, it is important to understand the discourses underlying how people with LBP in China explain the causes of their persistent or recurrent pain.

An in depth understanding of the beliefs about causes of persistent or recurrent LBP is argued to be helpful to tailor pain management [23, 41], and may be a critical element to reduce the burden of LBP. The primary aim of this study was to explore the discourses underlying the beliefs of people in China about what causes their persistent or recurrent LBP. The secondary aim was to investigate the individual’s perception of the information sources of these discourses.

## Methods

### Study design

This study used a cross-sectional online survey which was based on an earlier Australian survey exploring LBP beliefs [30]. The study was approved by the scientific and ethical review board of Vrije Universiteit Amsterdam (VCWE, number 2019-065R1). All participants provided online informed consent.

### Survey

The survey (see Additional file 1) was designed in collaboration with the researchers who conducted the Australian survey [30]. The survey was translated into Chinese by one of the researchers (YJL) who is a native speaker in Chinese and fluent in English. The translation involved direct translation of most of the words, but cultural adaptation where necessary. The appropriateness of the translation was verified and confirmed by a second Chinese native speaker fluent in English. The Chinese survey was pilot tested with four Chinese people (*n* = 2 with a history of LBP and *n* = 2 who studied linguistics), which resulted in slight modifications of the original translation.

The survey had three sections:

1. Background information: Participants were asked demographic questions and questions about their LBP characteristics (e.g., duration of LBP, intensity of LBP on 10-point numeric rating scale, usage of pain medication (Yes/No), presence of comorbidities (Yes/No - if the answer was Yes, participants were asked to list their comorbidities), absenteeism due to LBP (Yes/No - if the answer was Yes, participants were requested to describe the duration of their absenteeism), and impact of LBP on daily life (Yes/No)).
2. An open-ended question (Question 17) to explore patients’ perspectives of what they believed caused their persistent or recurrent LBP: What is your perception of why your low back pain is persistent or recurrent? Please kindly explain your answers. [您觉得自己的腰痛为什么会长时间持续或者不断复发呢?请您耐心的解释您的答案。]
3. A question (Question 18) to identify where these perspectives came from: Where does the perception listed above come from: (several options are possible) [您觉得上一题 您的看法来自:(可以多选)] 1) Healthcare professionals [医生或者其他医疗工作者]; 2) Internet [网络]; 3) Family [家人]; 4) Friends [朋友]; 5) Religion [宗教]; 6) Other [其他] __________________.

The Chinese language online survey was uploaded to a professional online questionnaire platform (WenJuanXing (问卷星)), and released through WeChat (微信) in Mainland, China. WeChat is the equivalent to the combination of WhatsApp and Twitter. The link to the survey was first shared in WeChat via researcher YJL’ s personal contacts with lay people and health care providers (physicians, physiotherapists) working in primary medical services and hospitals in China. Subsequently, the survey was further spread through these connections. Responses were translated back into English by a Chinese researcher (YJL) and checked for accuracy by a second Chinese reviewer. Discussion to consensus was used to resolve translation discrepancies.

### Participants

Participants were invited through WeChat (微信), the most popular Chinese social media platform. Inclusion criteria were: 1) aged between 18 and 65 years; 2) persistent or recurrent LBP within the last 6 months; and 3) proficiency in Chinese language. Participants were able to access the survey only if they responded affirmatively that, within the last 6 months, they were experiencing or had experienced LBP for more than 3 months.

### Sample size

Based on previous study experiences with satisfying the principal of theoretical saturation [30], we predicted that approximately 130 participant responses were needed to identify the range of discourses underlying pain beliefs in a Chinese population with persistent or recurrent LBP.

### Data analysis

The survey data in response to the question about causes of LBP were analysed using discourse analysis. Both the discourse and conventional content analysis (below) were inductive which means that no pre-existing theory was imposed on the analysis. Discourse analysis is a qualitative research methodology which considers that language constructs social and psychological reality [42]. This means that the language people use provides insights into how people view and act in the world [42]. For instance, people with LBP who claim that a herniated disc is the cause of their back pain, are considered to have a biomedical discourse underpinning their beliefs about the cause of their LBP. Drawing on these concepts, discourse analysis was used in this study to interrogate the underlying discourses behind the responses given by Chinese LBP population.

Three researchers (JS, GGMSP and YJL) reviewed the entire dataset independently and proposed potential discourses underpinning the data during a meeting. Subsequently, two researchers (YJL, GGMSP) formulated five tentative discourses in a consensus meeting. The five tentative discourses were reappraised by one researcher (JS). During a second consensus meeting, the five discourses were refined, and consensus was reached between the three researchers (JS, GGMSP and YJL). Subsequently, YJL and GGMSP independently reviewed the full dataset again and coded each participant’s response into one or more of the five discourses. Initial agreement about the coding was calculated between YJL and GGMSP. Discrepancies were discussed until consensus was reached between the two researchers. Finally, the complete dataset, the coding and the findings of the discourse analysis were reviewed by JS who agreed with the discourses and coding.

The data from the final question about where participants’ beliefs came from was analysed using descriptive statistics on the quantitative data (option 1 to 5) and conventional content analysis [43] on the qualitative data from option 6 ‘Other’. In cases where there were overlaps of the answers, the two types of data were compared. This type of analysis was able to provide a descriptive overview of where participants believed the discourses came from. Conventional content analysis was performed by two independent researchers (YJL and GGMSP) and discussion was used to reach consensus.

Sociodemographic and clinical information was analysed using descriptive statistics in SPSS version 24 (IBM Corp, Armonk, New York, USA). Normality of continuous variables was visually inspected by histograms, Q-Q plots and box plots. Also, Z-values of skewness and kurtosis, and the Kolmogorov-Smirnov tests (*n* > 50) or Shapiro-Wilk tests (*n* < 50) were performed. If the data were normally distributed, means and standard deviations were reported. Otherwise, medians and interquartile range were presented.

To observe whether there were any differences between participants with and without missing data, the main baseline characteristics were statistically tested with independent sample t-tests or Mann-Whitney U tests.

## Results

**Fig. 1:**
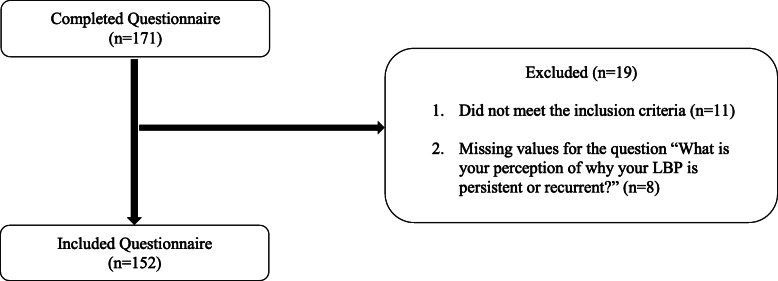
Flowchart of the study

There were no differences in sociodemographic and clinical data between participants with and without missing values. In total, 171 participants agreed to participate in the study. Nineteen participants were excluded as they did not fulfil the selection criteria or had missing values for question 17 (Fig. 1). A total of 152 responses were included for data analysis. Seventy-three percent (73%) of the study population was female, 98.7% were Chinese nationals and most came from South Central China (51.3%), which is also the second most populated region [44] . Fifteen percent indicated that they experienced LBP every day and the mean (SD) pain intensity was 3.9 (1.5) on a 10-point numeric rating scale. Additional participant characteristics are presented in Table 1.

**Table 1:** Participant characteristics (*n* = 152)

Age (years)	
Median (IQR)	29.0 (24.0–40.8)
Sex (% female)	73.0%
Nationality (%)	
Chinese	98.7%
Dutch	1.3%
Region	
South Central China (28.4% of total population)	51.3%
East China (29.5% of total population)	21.1%
North China (12.5% of total population)	15.1%
Southwest China (14.5% of total population)	7.9%
Northeast China (7.7% of total population)	3.9%
Northwest China (7.4% of total population)	0.0%
Other	0.7%
Years since the first episode of LBP^a^	
Median (IQR)	3.0 (0.2–6.0)
LBP everyday (% yes)	15.1%
LBP present now (% yes)	44.1%
Pain intensity^b^ (if LBP present now)	
Mean (SD) on 10-point numeric rating scale	3.9 (1.5)
Pain medication use in history (% yes)	5.9%
Comorbidities (e.g., cardiovascular diseases, arthritis)	
% yes	17.1%
Work/school absenteeism due to LBP (% yes)	17.1%
Duration of absenteeism^c^ (Days)	
Median (IQR)	14.0 (7.0–32.5)
LBP impacts daily life (% yes)	36.8%

Years since the first episode of LBP^a^ (11 missing values); Pain intensity^b^ (1 missing value); Duration of the absenteeism^c^ (5 missing values)

### What is your perception of why your low back pain is persistent or recurrent?

Five discourses were identified. Most participants answered this question with one or two sentences, and their responses were assigned into one or more of the identified discourses. The initial agreement between YJL and WSP was 90%, and all discrepancies were resolved through discussion. An overview of the five discourses is presented in Table 2 and below.

**Table 2:** Discourses identified from the answers to the question “What causes your persistent or recurrent LBP”

Discourses (Patterns of ideas) and n (%)	Explanation	Examples
LBP as biomedical problem. *N* = 101 (66.4%)	LBP is explained by incorrect postures, damage, degeneration or weakness of neuromusculoskeletal structures. Pain is produced or provoked because something is biomedically wrong in the body.	Participant 20: “Pelvic tilt anteriorly, lacking strength in abdominal muscles with hypertonic erector spinae muscles, thorax vertebrae kyphosis, slight scoliosis, flat foot problem and gait problem”
LBP as unbalanced lifestyle. *N* = 74 (48.7%)	LBP as a warning sign, or consequence, of an unbalanced lifestyle. A metaphorical individualised “balance scale” about the amount of exercise, sitting, standing, walking time or load.	Participant 21: “… Doing too much or too little exercise? ...” Participant 58: “Unregular daily routine …”
LBP is about menstruation and ‘kidney’ status. *N* = 14 (9.2%)	In Chinese culture, menstruation is important to women’s overall health while ‘kidney function’ is vital to men’s. Women during menstruation are generally believed vulnerable and emotionally unstable while men who have poor kidney function, are believed to have low sexual performance.	Participant 21: “ShenKui (kidney deficiency) ...” Participant 37: “Because of menstruation”
LBP is about the ‘Five Elements’ imbalance. *N* = 12 (7.9%)	There are ‘Five Elements’ in Chinese medicine: Water, Fire, Wood, Metal and Earth. It is considered important to keep the balance between these elements to maintain good health. Climate conditions such as wind, heat, dampness, dryness and cold are represented separately by wood, fire, earth, metal and water. It is thought that climate conditions can ‘invade’ the body and cause an imbalance in the Five Elements.	Participant 38: “… after staying in cold water for several hours, my back pain suddenly occurred” Participant 61: “Accumulation of Wind, Damp” Participant 114: “… and drinking too little water will cause my recurrent low back pain”
LBP is about energy status. *N* = 9 (5.9%)	Thinking low or disturbed energy status is a cause of LBP. In traditional Chinese medicine a low (disturbed) mental energy status can be caused by, or causes a possible ‘Qi’ stagnation, resulting in muscle pain. ‘Qi’ understood as a ‘matter-energy’ or ‘vital force’, connects physical and mental energies in individuals.	Participant 10: “JingShen status is not optimal” ‘Jing’ means essence while ‘Shen’ means ‘Mind’. ‘JingShen’ status can be understood as mental energy status. Participant 70: “… also because of life stress and fatigue caused by work”

Participant’s response can be coded into one or more of the five discourses

#### Discourse 1: LBP as a biomedical problem

‘LBP as a biomedical problem’ was the most prevalent discourse. Two-thirds of the responses were underpinned by this discourse. Participants explained their persistent or recurrent LBP by *physical damages, incorrect posture, muscle imbalance and congenital issues.* Their responses indicated that they considered their body in an anatomical, biomechanical and/or physiological way. Their underlying belief seemed to be that if there was something wrong biomedically in their back (body) that this would produce or provoke pain. For instance, some discussed physical damages as the cause of their ongoing LBP, such as Participant 16, who wrote: “I had an injury during long jump in secondary school and I didn’t pay attention to it. So, I probably got LaoSun (muscle strain) for a prolonged period”. ‘LaoSun’ is a common Chinese term which means overuse of muscles that can lead to muscle strain and injury [45]. Participant 72 wrote:

Probably because of continuous stimuli, and I didn’t get the right diagnosis and treatment for my low back injury. In the beginning, I had an injury in another part of the body which caused poor posture during running. Later on, this led to unequal left and right muscle strength which compressed and pushed out my vertebra.

Like a number of other participants, Participant 106 also discussed posture, attributing their LBP persistence or recurrence to “Lower-crossed syndrome, anterior tilt of my pelvic causes incorrect posture during standing”. And participant 157 wrote: “… Working posture is not correct”.

Also underpinned by biomedical discourses, one participant indicated congenital issues as the source of their LBP. Participant 98 wrote: “I had an X-ray, the doctor said there is a deformation in my bone, my family members also have hereditary low back problems”.

#### Discourse 2: LBP as unbalanced lifestyle

‘LBP as unbalanced lifestyle’ was the second most commonly reported discourse. Around half of participants’ answers were identified to fit within this discourse. LBP was described as a warning symptom or the result of an unbalanced lifestyle. A figurative individualised ‘balance scale’ that considers exercise, sitting, standing, walking time or load, could be identified from the responses. For instance, Participant 5 wrote: “I don’t perform enough exercise and physical activities, I sit for a long time” and Participant 21 thought the cause could be “Doing too much or too little exercise?”

Often, ‘LBP as unbalanced lifestyle’ seemed to be related to the first discourse ‘LBP as a biomedical problem’. For example, a quote selected from the response of Participant 59:

I think it’s because I’m not exercising regularly. When I do go to gym, I will definitely train my lower back muscles with the back-extension equipment. The problem is, I should have gone more frequently than I usually do.

First, this participant pointed out the importance of balanced lifestyle (exercising regularly). They then related the lack of exercise to the idea that back muscles should be trained specifically (indicating a biomechanical view of LBP). The participant then reinforced the importance of a balanced lifestyle by adding “I should have gone more frequently (to the gym) than I usually do”. One response related to unbalanced lifestyle based on traditional Chinese beliefs regarding pregnancy: “I didn’t take care of my low back during ZuoYueZi (postpartum care)” (Participant 143). This answer related to traditional Chinese health beliefs. ZuoYueZi is a part of Chinese custom that intends to improve health after pregnancy [46]. After delivery, it is strongly recommended that the mother takes particular actions such limiting movement, eating special food and not washing her hair [46]. Also, family members are an important part of ZouYueZi, providing social support to the mother, such as doing housework and taking care of the baby [46].

#### Discourse 3: LBP is about menstruation and ‘kidney’ status

‘LBP is about menstruation and kidney status’ was the third identified discourse. Approximately, 9% of the responses appeared from this discourse. In Chinese culture, menstruation is important to women while ‘kidney function’ is vital to men. To a certain extent, menstrual function and ‘kidney function’ reflect reproductive health. The understanding of ‘kidney function’ in Chinese medicine is fundamentally different to Western medicine [47]. In traditional Chinese medicine, the kidney is not be considered as a real organ but as a symbol that controls reproductive health [40] and causes LBP when the kidney is deficient [40].

As is evident in this quote from Participant 50, “Because of menstruation, I have LBP 2 days before menstruation, but I don’t know the reasons behind it”, several participants considered menstruation to be the cause (or one of the causes) of their LBP. Further, Participant 28 wrote, “LBP appears before menstruation” and Participant 37 responded, “Because of menstruation”. One male participant indicated “ShenKui” was a reason for his LBP. ‘Shen’ means Kidney while ‘Kui’ means deficiency [48]. In China, women are generally believed vulnerable and emotionally unstable during menstruation [49], whereas men who have ShenKui are believed to have low sexual performance [48]. Psychosocial stresses in this discourse may be relevant to the LBP reported by participants.

#### Discourse 4: LBP is about the ‘five elements’ imbalance

The discourse ‘LBP is about Five Elements imbalance’ was only occasionally mentioned (7.9%) but was identifiable from the responses. Related to the balance of Yin and Yang, there are ‘Five Elements’ in Chinese medicine: Water, Fire, Wood, Metal and Earth [40]. There is a self-regulating balance within the five elements. For example, Water balances Fire, but Fire produces Earth that balances Water in return. In traditional Chinese medicine it is considered important to encourage this self-regulating balance to maintain good health. Water is considered to be the foundation of the other Elements [40]. This appears to be the discourse underpinning Participant 114’s response “Drinking too little water will cause it (LBP) to recur”.

The Five Elements can represent different seasons, directions, colours, tastes and climates [40]. For example, spring, summer, autumn and winter are represented by wood, fire, metal and water, respectively [40]. Climates such as wind, heat, dampness, dryness and cold are represented separately by wood, fire, earth, metal and water, respectively. The self-regulating balance can be disturbed by exterior invasion of cold, wind and dampness which is believed to cause LBP [40]. Also, external cold can affect ‘kidney function’ when it invades the low back region, which often happens to modern women due to exposure of lower abdominals and loins in modern fashion [40]. Thus, participant’s answers related to climates (e.g., “Probably suffer from cold” - Participant 9), and seasonal change (e.g., “… Pain will certainly occur during autumn-winter seasonal rotation and spring-autumn seasonal rotation, mainly because of climates …” - Participant 41) were considered to be underpinned by this ‘Five Elements’ discourse.

#### Discourse 5: LBP is about energy status

The least common discourse (5.9%) was based on ‘LBP is about energy status’. In traditional Chinese medicine, ‘Qi’, understood as a ‘matter-energy’ or ‘vital force’, connects physical and mental energies in individuals [40]. Qi should circulate freely inside the body and also flow in and out the body in a healthy situation [40]. A low (disturbed) energy status can be caused by, or causes, ‘Qi’ stagnation, resulting in muscle pain [40]. Answers related to low energy status were considered to draw from this discourse. For example, Participant 10 responded, “… JingShen status is not optimal”. ‘Jing’ means ‘essence’ while ‘Shen’ means ‘mind’. ‘JingShen’ status can be understood as energy status. Also, answers indicating a disturbed energy status, caused by stress or mental fatigue, were underpinned by this discourse. For example, Participant 70 wrote, “… also because of life stress and fatigue caused by work”.

### Where does the perception come from?

Most participants selected only one of the five options and did not provide additional sources as ‘other’ options. Almost two thirds indicated that their perception of what causes their LBP to become persistent or recurrent came from healthcare professionals (*n* = 90, 59.2%). The options ‘internet’ (*n* = 37; 24.3%), ‘family’ (*n* = 35; 23.0%) and ‘friends’ (*n* = 25; 16.4%)) were also frequently listed. ‘Religion’ 0 (0%) was not indicated as an information source. Originally, the option ‘other’ was selected by 30 participants. However, two answers overlapped with the option ‘healthcare professionals’, and were moved from the option ‘other’ and into the option ‘healthcare professionals’. In total, 28 participants (18.4%) selected the option ‘other’. One of these reported two information sources and another reported three. Under the option ‘other’, most reported some sort of self-reflection (*n* = 24, 15.8%) as information source. Others indicated previous medical related education (*n* = 2, 13.2%), scientific literature (*n* = 1, 0.7%), and TV programmes (*n* = 1, 0.7%) as information sources. Three participants (2.0%) provided unclear answers. For example, Participant 19 answered “my personal experience” and Participant 37 wrote “daily observation”. An overview of responses for this question are provided in Table 3.

**Table 3:** Information sources identified from the answers to the question “Where does your perception come from?”

Source of beliefs	n (%)
Healthcare professionals	90 (59.2%)
Internet	37 (24.3%)
Family	35 (23.0%)
Friends	25 (16.4%)
Religion	0 (0%)
Other	Total^a^: 28 (18.4%)
*- Self-reflection*	*24 (15.8%)*
*- Education*	*2 (1.3%)*
*- Scientific literature*	*1 (0.7%)*
*- TV programme*	*1 (0.7%)*
*- Unclear answers*	*3 (2.0%)*

^a^Under the option ‘Other’, one participant reported two information sources and another one reported three

## Discussion

This study identified five key discourses underlying the beliefs of people living in China about what causes their persistent or recurrent LBP. The most predominant discourse was that LBP persisted or recurred due to biomedical problems, followed by the discourses influenced by traditional Chinese medicine related beliefs: unbalanced lifestyle, menstruation and ‘kidney’ status, the ‘Five Elements’ imbalance, and mental energy status. Most participants responded that their pain beliefs were based on information derived from healthcare professionals followed by the internet and family.

A similar study assessed discourses underpinning beliefs about the causes of the persistence of LBP in participants living in Australia [30]. Four discourses were identified in that study: 1) Body as a machine; 2) LBP as permanent/immutable; 3) LBP is complex; 4) LBP is very negative. ‘Body as a machine’ is comparable with the discourse ‘LBP as biomedical problem’ and was also the most common discourse in the Australian study. The assumed biomedical causes to explain the ongoing nature of LBP was thus a common trend in both the Chinese and Australian study populations. This comparable understanding of the body between Chinese people and Western people is not surprising. Since 1978 the ‘Reform and Opening’ policy has been embraced in China, and this has included an increasing uptake of Western medical methods and interventions [50]. Currently, the principal medical practice in China is Western medicine [50–52]. However, even in the two-thirds of participants living in China who explained the causes of their pain with biomedical discourse, almost half of them related the causes to other traditional Chinese medicine related discourses. Unlike the previous studies which reported the homogenous usage of biomedical model to explain chronic LBP in Western society [27, 28, 30] and potentially across cultures [35], in our study, at least in the case of LBP, people in China often think about their health beyond the biomedical paradigm, combining traditional Chinese medicine related beliefs to the aforementioned paradigm.

From the answers underpinned by the discourse *LBP as unbalanced lifestyle*, a metaphorical personalised ‘balance scale’ about time or load of exercise, sitting, standing or walking could be identified. The language the participants used to describe time or load, was ‘too much’, ‘too little’ and ‘too long’. However, there was no specific duration, number or load mentioned by the participants. This ‘balance scale’ seems personal. Although lifestyle causes of ill-health are also found in Western medicine, considerations of balance can be found in traditional Chinese medicine which has long discussed balance as a key to health [40, 53]. Any imbalance, e.g., the imbalance between rest and exercise, unbalanced emotion or diet, too much and too little work or sex, can become a cause of disease based on traditional Chinese medicine [40, 53]. The idea of an individualised balance scale is part of the Chinese culture. *LBP as unbalance lifestyle* from a Chinese perspective was at times entangled with the common discourse *LBP as a biomedical problem* as many participants related the time or load with certain positions or postures to explain their ongoing LBP. To a certain degree, the discourse *LBP as unbalanced lifestyle* may show how Chinese philosophy merges with a Western biomedical view.

The other three discourses regarding LBP as menstruation and ‘kidney’ status, the ‘Five Elements’ imbalance, and energy status were less common but unique. These three discourses appear to interconnect with one another. For instance, an explanation that the external invasion of cold can cause LBP from the discourse ‘the ‘Five Elements’ imbalance’, can be related to the discourse ‘menstruation and ‘kidney’ status’, because the invasion of cold is believed to be harmful to ‘kidney’ in traditional Chinese medicine [40]. Another example of interconnections is the idea that Qi stagnation can cause LBP from the discourse ‘energy status’. This concept might be related to the discourse ‘menstruation and ‘kidney’ status’, because Qi stagnation is also thought to result in irregular menstrual status and ‘kidney’ deficiency, which ultimately provokes LBP [40]. Importantly, these three discourses together with the previous discourse ‘unbalanced lifestyle’, eventually seem to be related to the overarching theme of ‘balance’ - the key concern of health discussed in traditional Chinese medicine [40, 53]. These four discourses likely demonstrate the ongoing impact of traditional Chinese medicine on contemporary Chinese society and represent the complexity of the population’s understandings of what causes their LBP to be persistent. The four discourses underlying Chinese people’s pain beliefs might indicate high thoughtfulness to the body, mind and environment which may generate related neural networks that collaborate to evoke pain [54] and increase susceptibility to pain. However, these discourses might guide people in China to improve self-management (e.g. adjusting unbalanced lifestyle or simply waiting menstrual cycle to pass), unlike Western chronic LBP patients often searching for specific medical diagnosis under the biomedical paradigm [28].

Reported by the earlier study [30], the Australian group also frequently indicated their LBP as permanent/immutable and very negative. The Chinese group seemed more positive about their persistent LBP. This Chinese group seemed more positive about their persistent LBP, compared to an earlier similar study [30] that Australian chronic LBP patients frequently indicated their pain as permanent/immutable and very negative. We reanalysed of the present data by reallocating Chinese participants’ responses into the discourses *LBP as permanent/immutable and LBP is very negative*. Only two Chinese participants indicated their LBP as permanent/immutable with possible negative emotions, by complaining about no useful LBP medical interventions available or saying LBP is inevitable and impacts one’s study and life. Although the reasons for these differences across the populations are not known, two possible explanations are that; 1) the study may represent a population with less severe symptoms than the Australian study; and 2) stoicism (meaning showing no emotions when encountering pleasure or pain) is considered as a positive trait in Chinese culture [55]. In contrast to our findings about patients living in China, other research suggests that Chinese healthcare professionals [56, 57] show higher levels of fear avoidance beliefs related to physical activities than Western physicians [58] and medical specialists [59], and Chinese nurses have pessimistic views about LBP as many nurses experiencing LBP planned to quit their job due to their back pain [56]. However, we suggest that these comparisons [56–59] should be viewed with caution as those other studies used variables developed from Western ways of thinking to quantify pain-related beliefs [34]. Moreover, the overuse of MRI in diagnosing LBP in Chinese hospitals may imply that Chinese care providers are grounded in biomedical causes for LBP [13].

Our study found that healthcare professionals were the main (59%) information source of beliefs about causes of persistent or recurrent LBP in Chinese group. This finding was much lower than the previous Australian study where 89% of participants attributed “healthcare professionals” as information source [30]. Information sources ‘Family’ and ‘Friends’ were higher (23.0 and 16.4%) in the Chinese sample, compared with 9.0 and 5.0% respectively in the Australian sample [30]. These differences might be explained by a less severe LBP population in our study. Only 15.1% of Chinese versus 82.0% of Australian participants reported having daily LBP. Due to the less severe level of LBP, we assume that less participants needed to seek medical professionals’ help. A re-analysis including only the participants who reported LBP every day (*n* = 23), revealed that 52.2% of Chinese people with LBP every day, selected ‘healthcare professionals’ as information source. This percentage is still lower than in the Australian study [30]. However, these comparisons should be interpreted with caution due to the small sample size. The lower percentage of ‘healthcare professionals’ information source, might also be explained by Chinese people’s historical grounding in Confucianism, which emphasises family and community needs over those of an individual. As health concerns are viewed as family problems rather than personal ones, seeking help from healthcare professionals may be considered a shameful revelation of private family matters to outsiders [55]. This might be one reason why less Chinese participant chose to seek medical help, even if they had LBP every day. Confucianism has been identified as a cultural barrier to the Chinese population receiving pain interventions [55]. However, Chinese healthcare professionals have been held negative beliefs about LBP [56, 57] and could negatively influence pain related beliefs of patients. Confucianism might be considered positive for people in China with LBP by avoiding unhelpful medical help.

It is important to consider the representativeness of this study population. Notably, half of the participants were from South Central China and most other participants were from either East China or North China. This can be explained by the way the data were collected by WeChat as most connections were with South Central, East and North region. Due to the lack of data from less-developed China, the study findings may not be as representative of those parts of China. Moreover, pain beliefs influenced by culture are hard to separate from socioeconomic factors [34, 60]. As we did not gather participants’ socioeconomic status or educational level in this study, our study findings should be interpreted with caution with respect to representativeness. Considering also to the demographics of this study population with 73% females and generally mild LBP, the results may not be as applicable to populations with predominantly males and people with more severe conditions*.* Additionally, due to our data collection methods, the representativeness of the current study might also be focussed towards younger and richer people with LBP, as they are more likely to have access to mobile phones, internet and social media.

Although we have compared some of our results to an Australian study [30] upon which we based our survey questions, these comparisons need to be interpreted with caution as there were some necessary changes to the wording in line with the cultural translation approach that we used. We did not translate the survey according to the recommended guidelines for translating questionnaires [61]. As the questions were very straightforward, there was little room for misunderstanding, but translational errors cannot be ruled out. We have also attempted to address any potential issues by not overstating the comparisons between the findings of the two studies.

The different belief system of LBP population in China found in our study, supports research that suggests that cultural factors influence pain-related factors [19, 33, 34, 60, 62]. For example, in contrast with the overwhelming LBP medical care in the West [14, 25, 32], a Nepalese study showed that people who suffered from LBP continued with their daily activities without seeking medical help as they consider LBP to be a normal aging process [60]. Insights from different cultural perspectives can provide useful information to understand patients’ beliefs about the causes of pain and can therefore assist with tailoring treatments and addressing beliefs.

Our study is the first study that examined the discourses underpinning pain beliefs in a Chinese population with persistent or recurrent LBP. It demonstrates the complexity of the Chinese population’s pain beliefs which are beyond the biomedical paradigm. The complexity of this way of thinking about pain is largely influenced by the concept of ‘balance’ from traditional Chinese medicine. This study also provides a starting point for future research in pain management. It is recommended for future research that customised interventions to explain LBP appropriately, should contain biomedical and thinking of psychosocial causes with integration of Chinese culture related thoughts concerning causes of LBP. Integrating the concept of ‘balance’ into modern neuroscience pain education could be helpful for the future management of LBP in China.

## Conclusions

The findings of this study support those from previous studies in other cultural groups that suggest that people frequently use biomedical viewpoints to explain the persistence or recurrence of their LBP. This perspective appears to be in Chinese populations as well. However, it also establishes the persistent impact of traditional Chinese medicine on contemporary Chinese society’s understandings of LBP mainly related to the concept of ‘balance’. Findings suggest that comprehensive LBP management for Chinese people should be culturally relevant, multifaceted and involve healthcare professionals, family and the patient.

## Supplementary information

**Additional file 1.** The survey. The English and Chinese version of the survey.

## Data Availability

The datasets used and/or analysed during the current study are available from the corresponding author on reasonable request.

## Abbreviations

LBP: Low back pain
YLDs: Years lived with disability
