# Supplementary material for: How do people in China think about causes of their back pain? A predominantly qualitative cross-sectional survey
Source: BMC Musculoskelet Disord. 2020 Jul 21;21:476. doi: 10.1186/s12891-020-03500-1 (PMC7372862; doi:10.1186/s12891-020-03500-1)
Supplement: Supplementary file 1 — Additional file 1. The survey. The English and Chinese version of the survey. [file 12891_2020_3500_MOESM1_ESM.docx]

**Additional file 1 - The survey**

**English version**

**QUESTIONS**

1) Age in years:

2) Gender:

Male

Female

3) Nationality:

Chinese

Other ________

4) In which province do you come from?

5) How long ago did your first experience LBP? (Specify the number in days, weeks, months or years)

6) Do you experience LBP every day?

Yes, I experience LBP every day.

No, I don’t experience LBP every day.

7) How many days per week/month/year do you have low back pain?

8) What is the time frame that your LBP varies?

My back pain changes every day.

My back pain changes every week.

My back pain changes every month.

No, my back pain does not change.

Other ________

9) Do you have periods without LBP?

Yes, I have period without LBP.

No, I don’t have period without LBP.

10) If yes, what would you consider to be the average duration of your period without LBP? (e.g. number of hours, days, weeks etc)

11) Do you experience back pain now?

Yes, I am in pain now.

No, I am not in pain now.

12) If you experience back pain now, how would you rate your LBP intensity now from 0 to 10.

(0 means no pain, and 10 means the worst possible pain)

| ○0 | ○1 | ○2 | ○3 | ○4 | ○5 | ○6 | ○7 | ○8 | ○9 | ○10 |
| --- | --- | --- | --- | --- | --- | --- | --- | --- | --- | --- |

13) Do you use pain medication for LBP?

Yes, I use. (please describe the type and doses of the medication you use) ________

No, I don’t use pain medication.

14) Do you have other diseases/disorders beside your LBP (e.g. high blood pressure, heart diseases, joint problems)?

Yes, I have. (Please name the diseases/ disorders you have) ________

No, I don’t have other diseases/ disorders.

15) Did you have work (school) absenteeism due to LBP?

Yes, I had work (school) absenteeism due to my back pain. (Please describe for how long in weeks) _________

No, I don’t have work (school) absenteeism due to my back pain.

16) In general, does your low back pain have an impact on your everyday life?

Yes

No

17) What is your perception of why your low back pain is persistent or recurrent? Please kindly explain your answers in a text.

18) Where does the perception above come from? (More options are possible)

Healthcare professionals

Internet

Family

Friends

Religion

Other ________

**If you would like to receive the summary of this study, please write down your e-mail here: ________________**

**Chinese version**

**问题**

1）年龄(年)：

2）性别

| 男 |
| --- |
| 女 |

3）国籍

| 中国 |
| --- |
| 其他 ________ |

4) 您来自哪个省份？

5) 您第一次腰痛发生在多久之前？(请以天，周，月或者年计算。例如，我第一次腰痛发生在大约3周前。)

6) 您每天都经历腰痛吗？

| 是的， 我每天都腰痛。 |
| --- |
| 不是，我不是每天都腰痛。 |

7) 请您估计**每周(月或者年)**有**几天**腰痛？(例如，我每周大约有1天腰痛；我大概每个月有3天腰痛。)

8) 您腰痛变化的模式是什么样的？ (变化可理解为，疼痛增加或者减少，疼痛的区域发生改变…)

| 我的腰痛每天都变化。 |
| --- |
| 我的腰痛每周发生改变。 |
| 我的腰痛每月发生改变。 |
| 没有，我的腰痛从来不发生变化。 |
| 其他 ________ |

9) 您有过腰不痛的时间段吗？ [单选题] *

| 有， 我有过腰不痛的时间段。 |
| --- |
| 没有，我没有过腰不痛的时间段。 |

10) 如果上一题的回答是 “有”，请您估计腰不痛的时间大约是多少？(比如，多少小时，多少天或者多少周…)

11) 您现在腰痛吗？

| 是的，我现在腰痛。 |
| --- |
| 不是的，我现在腰不痛。 |

12) 如果您现在腰痛，您疼痛的级别是什么样的呢，从0到10？（0代表无痛，10代表最无法忍受的疼痛）

| ○0 | ○1 | ○2 | ○3 | ○4 | ○5 | ○6 | ○7 | ○8 | ○9 | ○10 |
| --- | --- | --- | --- | --- | --- | --- | --- | --- | --- | --- |

13) 您使用止疼药吗？

| 是的，我使用(请写下药的类型和剂量) ________ |
| --- |
| 不是的, 我没有使用止疼药。 |

14) 除开腰痛，您患有其他疾病吗？ (比如，高血压，心脏病，关节病…)

| 是的，我患有其他疾病。(请写下您所患有的疾病) ________ |
| --- |
| 不是的，我没有患有其他疾病。 |

15) 您有因为您的腰痛，误工(学)过吗？

| 是的，我有因为腰痛误工(学)过。(请写下误工（学）的时间，以周计算) ________ |
| --- |
| 不是的，我没有因为腰痛而误工(学)过。 |

16) 总体来说，您觉得的您的腰痛影响你每天的生活吗？

| 影响 |
| --- |
| 不影响 |

17) 您觉得自己的腰痛为什么会长时间持续或者不断复发呢？(请您耐心的解释您的答案。)

18) 您觉得上一题您的看法来自(可以多选):

| 医生或者其他医疗工作者 |
| --- |
| 网络 |
| 家人 |
| 朋友 |
| 宗教 |
| 其他 ________ |

**如果您想收到此研究的结果，请留下您的邮箱: ________________**
